# PD53 - Budesonide therapy reduces sCD30-levels in atopic infants with recurrent respiratory symptoms

**DOI:** 10.1186/2045-7022-4-S1-P53

**Published:** 2014-02-28

**Authors:** Anne Kotaniemi-Syrjänen, Weronika Delezuch, Kristiina Malmström, Pekka Malmberg, Anna Pelkonen, Kari Punnonen, Irma Matinlauri, Mika Mäkelä

**Affiliations:** 1 HUCH Skin and Allergy Hospital, Helsinki, Finland; 2 ISLAB Laboratory Centre, Kuopio, Finland; 3 HUSLAB, Helsinki University Central Hospital, Helsinki, Finland

## 

Only minority of infants with recurrent or persistent respiratory symptoms will develop asthma. The development of asthma is associated with Th1- and Th2-cell activity imbalance. Increased levels of Th1 activation marker, serum soluble CD26 (sCD26), and Th2 activation marker, serum soluble CD30 (sCD30) have been found in asthmatic adults and school children. Whether these markers are of clinical use in infants with troublesome lung symptoms has not been evaluated before.

**Figure 1:**
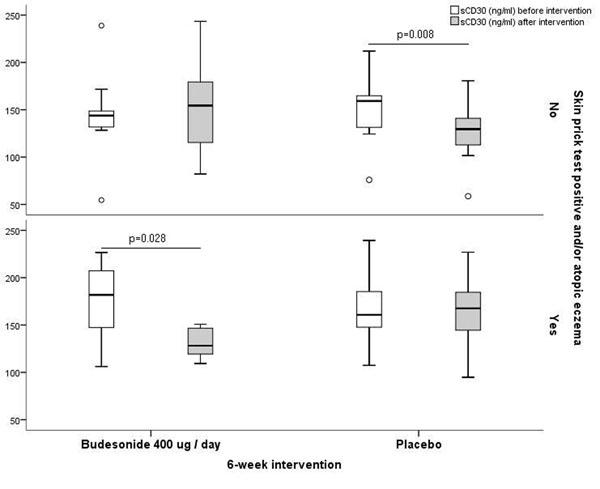


Altogether 43 children aged 4 to 26 months with persistent cough, recurrent wheeze, or shortness of breath were investigated clinically and randomized to receive either budesonide 400 ug/day or placebo for 6 weeks. There were no differences in baseline data like gender, atopic traits, clinical findings, or whole body plethysmography measurements between these two intervention groups. At baseline, serum sCD26 and sCD30 levels did not differ between the intervention groups. After the 6-week intervention there was a significant decrease in sCD30 levels in atopic (i.e. skin prick test positive and/or having atopic eczema) children treated with budesonide (p=0.028), and in non-atopic children who received placebo (p=0.008), but no changes in sCD30 levels in atopic children treated with placebo (p=0.753), or in non-atopic children treated with budesonide (p=0.695) (Fig.1). As regards sCD26, a significant increase was seen between the levels measured before and after the 6-week intervention (p=0.012), but the increase was independent of the type of the intervention.

In conclusion, Th1-type immunity is suppressed and Th2-type immunity activated in infants with troublesome lung symptoms. However, only atopic infants may benefit of anti-inflammatory inhalants.

